# Supplementary material for: A retrospective characterization of pediatric facemasks marketed in the United States and implications for future designs
Source: PLoS One. 2024 Sep 19;19(9):e0307879. doi: 10.1371/journal.pone.0307879 (PMC11412539; doi:10.1371/journal.pone.0307879)
Supplement: S1 File — (DOCX) [file pone.0307879.s001.docx]

**S1** **Supporting Information.**

**S1 Text. Literature Review of Breathing Flow Rate in Pediatric Population**

**Keywords:** pediatric + breathing flow rate, pediatric + inhalation flow rate.

Roy and Courtay mentioned breathing flow rates for different ages, considering both light and heavy exercise. Additionally, for older ages, they provided separate values for males (M) and females (F) (S1 Table) [1]. Based on this reference the maximum flow rate in 12-18 year age group is 40 – 50 LPM for heavy breathing.

**S1 Table.**  **Breathing Flow rates as reported by Roy and Courtay [1].**

| **Age (years)** | **Inspiratory Flow Rate (LPM)** | | |
| --- | --- | --- | --- |
|  | **Sitting Awake** | **Light exercise** | **Heavy exercise** |
| Newborn | - | 1.5 | - |
| 1 | 3.6 | 5.78 | - |
| 2 | 3.9 | 6.3 | - |
| 4 | 4.9 | 8 | - |
| 5 | 5.3 | 9.5 | - |
| 6 | 5.8 | 11 | 19 |
| 8 | 6.45 | 17 | 28 |
| 10 | 6.3 | 19 | M: 37 F: 31 |
| 12 | M: 7.8 F: 6.5 | M: 21 F: 19 | M: 40 F: 36 |
| 14 | M: 8.1 F: 6.7 | M: 23 F: 20 | M: 45 F: 42 |
| 15 | M: 7.95 F: 6.6 | M: 23 F: 22 | M: 49 F: 43 |
| 16 | M: 8.7 F: 7 | M: 25 F: 22 | M: 50 F: 45 |
| 18 | M: 10 F: 7.05 | M: 26 F: 22 | M: 51 F: 45 |

Xi et al., based on published respiratory parameters, reported the inhalation flow rates under quiet breathing to be 3.8 LPM for the 10-day-old girl, 6.5 LPM for the 7-month-old girl, 11.2 LPM for the 5-year-old boy, and 18 LPM for the adult. They also investigated particle deposition in the lungs for breathing scenarios ranging from sedentary to heavy activity conditions (i.e., 2–45 LPM) but did not specify the flow rate for heavy activities at 5-years [2]. However, in another study, they considered inhalation flow rates for a 5-year-old child ranging from sedentary (3 LPM) to heavily active (30 LPM) conditions. [3].

Kesavan, J., et al., reported breathing flow rates for three different age groups considering different activities (S2 Table) [4].

**S2 Table. Breathing Flow rates as reported by Kesavan et al. [4]**.

| **Age (years)** | **Inspiratory Flow Rate (LPM)** | | | |
| --- | --- | --- | --- | --- |
|  | **Sitting Awake** | **Light Activity** | **Light Exercise** | **Heavy Exercise** |
| 2 | 2.5 | 5 | 7.3 | 10.2 |
| 5 | 4 | 8 | 9.5 | 13.3 |
| 18 | 10 | 15 | 25 | 35 |

Further research was also conducted to assess the typical flow rates used in devices used for respiratory support. Ji-Won Kwon discussed the recommended flow rates in pediatric patients, as outlined in S3 Table [5]. Ejiofor et al. provided information about flow rates for different ages and weights, which can be found in S4 Table [6]. Lodeserto et al. indicated pediatric breathing flow rates of 2 L/kg/min [7]. Utilizing this formula yields flow rate values that are almost consistent with those reported in S3 and S4 Tables. The Royal Children's Hospital Melbourne specified a flow rate of 2L/kg/min for patients up to 12kg, with an additional 0.5L/kg/min for each kilogram above 12kg (up to a maximum of 50 LPM) [4]. These flow rates appear to be consistent with the flow rates typically observed for heavy breathing exercises as well.

**S3 Table. Breathing Flow rates as reported by Ji-Won Kwon [5]**.

| **Age** | **Body weight (kg)** | **Flow range (LPM)** |
| --- | --- | --- |
| ≤ 1 month | < 4 | 5-8 |
| 1 month - 1 year | 4-10 | 8-20 |
| 1-6 yeas | 10-20 | 12-25 |
| 6-12 years | 20-40 | 20-30 |
| 12-18 years | > 40 kg | 25-50 |

**S4 Table. Breathing Flow rates as reported by Ejiofor et al. [6].**

| **Age** | **Body weight (kg)** | **Flow range (LPM)** |
| --- | --- | --- |
| 1 month | 4 | 6-10 |
| 6 months | 8 | 8-12 |
| 1 year | 10 | 15-25 |
| 3 years | 15 | 20-30 |
| 6 years | 20 | 25-45 |
| 12 years | 35 | 30-50 |
| 18 years | 75 | 40-60 |

**S2 Text. Literature Review of Breathing Resistance in Pediatric Population**

**Keywords used in google scholar search:** pediatric + breathing resistance, pediatric + resistance in lungs, pediatric + respiratory resistance.

Cogswell reported airway resistance and total respiratory resistance in children aged from 2 to 17 years [8]. Airway resistance can be measured throughout the respiratory cycle using whole-body plethysmography. Total resistance of the respiratory system can be measured during relaxed expiration following end inspiratory occlusion. According to Cogswell's report, the airway resistance ranges from 0.67 to 1.17 mmH_2_O/LPM, and total respiratory resistance ranges from 0.83 to 1.83 mmH_2_O/LPM which is consistent with studies by Solymar et al. [9]. These resistance values show a trend with the height of the children, decreasing as height increases. Another study by Hudgel et al., reported respiratory resistance for a group of young students, ranging from 0.67 to 1.27 mmH_2_O/LPM during wakefulness and from 0.94 to 1.98 mmH_2_O/LPM at stage 2 non-rapid-eye-movement sleep [10].

Coyne et al. reported Inspiratory and Expiratory Resistance for a group of students at the University of Maryland, ranging from 0.37 to 2.1 mmH_2_O/LPM for Inspiratory Resistance and 0.15 to 0.30 mmH_2_O/LPM for Expiratory Resistance [11]. Adult parameters are also shown in S5 Table.

**S5 Table. Summary of the Reported Respiratory Resistance.**

| **Subject** | **Reported Respiratory Resistance (mmH_2_O / LPM)** | **Reference** |
| --- | --- | --- |
| Children, 2 to 17 years | Airway resistance 0.67 to 1.17  Total respiratory resistance 0.83 to 1.83 | Cogswell, 1973 |
| Children, 2 to 18 years | Inspiratory impendence: 2 kPa/L/s at 2 years and 0.3 kPa/L/s at 18 years which translates to 0.5-3.33 mmH_2_0/LPM across age range of 2-18 years. | Solymar et al, 1985 |
| Child, 5-year-old | Pressure drop 200 Pa at 30 LPM converted to mmH_2_O/LPM = 0.68 | Xi et al., 2012 |
| Young students | during wakefulness 0.67 to 1.27  during sleep 0.94 to 1.98 | Hudgel et al., 1993 |
| Group of students | Expiratory Resistance 0.15 to 0.30  Inspiratory Resistance **0.37** to 2.1 | Coyne et al., 2006 |
| Healthy individuals,  25-64 years. | Airway resistance 0.34 to 0.51 | Powell et al., 2012 |
| Adults | Maximum resistance in N95 respirators beyond which there is physiological discomfort 9 mmH20 | Roberg et al., 2013 |
| Breathing resistance measurements for adults | Total breathing resistance in lungs = 750 Pa or 73.4 mmH20 at 105 LPM flow rate | Xu et al., 2021 |

**S3 Text****. Oscillatory flow profiles of the QuickLung® Breather breathing simulator**


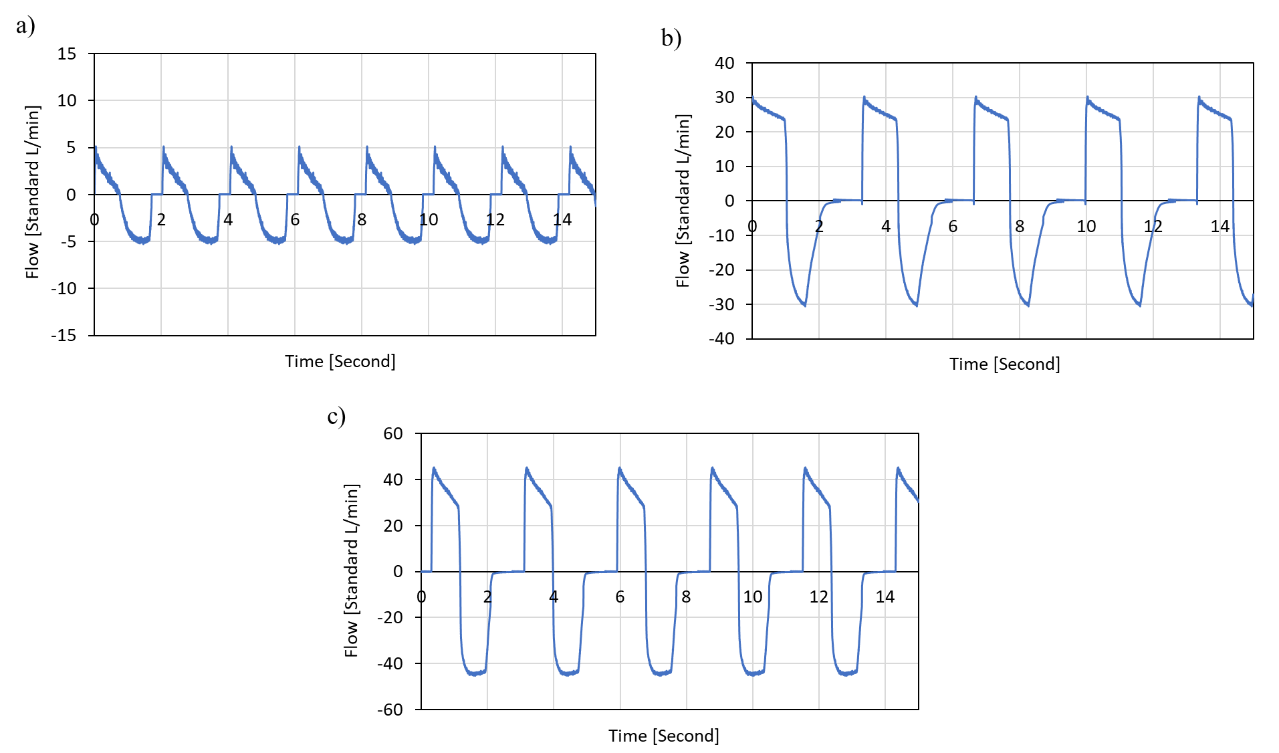


***S1 Fig.*** *The oscillatory flow profiles of the breathing simulator, a) ±5, b) ±30, and c) ± 45 LPM.*

**S4 Text. Literature Review of Facial Measurements in Pediatric Population**

**Keywords:** inter-pupillary distance + age, bizygomatic breadth + age, lower-face height + age, ear-sellion depth + age, stature + age, weight + age.

a. Inter-pupillary distance with age

Of all the facial measurements, the inter-pupillary distance has perhaps been most studied. Therefore, we included it as a metric for measurements. In a study by Filipović, 300 randomly selected healthy subjects, aged between 5 and 60 years, participated in an assessment of their inter-pupillary distance. Each age group consisted of 50 subjects. The mean inter-pupillary distance for subjects aged 5 to 10 was 5.1±1.5 cm. For subjects aged 11 to 20, it was slightly wider by 0.7 cm, measuring 5.8±2.5 cm. Interestingly, from the age of 20 to 60, the inter-pupillary distance remained constant at 6.3±2.3 cm (S2 Fig) [12].

***S2 Fig.*** *Inter-pupillary distance with age [12]. The corresponding measurements for the headforms have also been shown.*

In another study conducted by MacLachlan and Howland, normal interpupillary distances were measured in a population of 1311 subjects, ranging in age from 6 month to just over 19 years [13]. The study observed that, on average, interpupillary distances increased from 4.3 to 6.3 cm over the first 19 years of life. The relationship between average interpupillary distances and age in years was found to be approximated by second-order polynomial equations for both females and males.

In a study conducted by the Highway Safety Research Institute at the University of Michigan, Ann Arbor, various measurements were taken from over 4100 subjects [14]. These measurements included interpupillary distance, bizygomatic breadth, lower face height, ear-sellion depth (S3 Fig). The study aimed to represent, as closely as possible, the U.S. population with respect to race, demographics, and socio-economic factors. The deviation of the facial features of the manikins we have used is provided in S6 Table below.


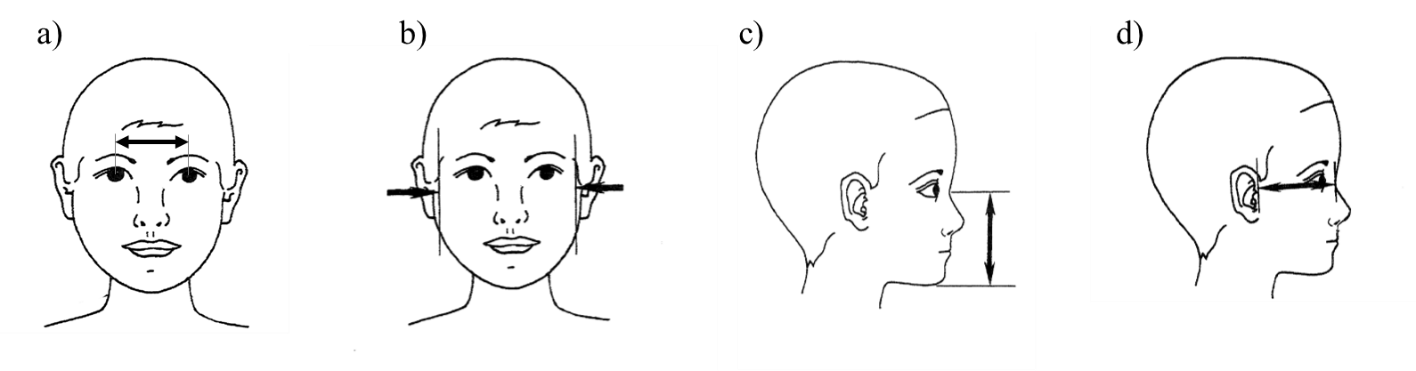


***S3 Fig.*** *Facial Dimensions: a) Interpupillary Distance, b) Bizygomatic Breadth, c) Lower Face Height, and d) Ear-sellion Depth [14].***S6 Table. Headforms Details and Deviation in Facial Anthropometrics from Human Subject**

| Headform | Age (year) | Gender | Reference | Facial anthropometric deviation from the human subject norm | | | |
| --- | --- | --- | --- | --- | --- | --- | --- |
|  |  |  |  | Interpupillary | Bizygomatic breadth | Lower face height | Ear-Sellion depth |
| Betty | 2 | Male | Dizzy headform reduced by 113%. | 3.0 % | -1.0 % | 1.2 % | -3.7 % |
| Roberta | 5 | Female | FDA Virtual Family Project [15] | -1.9 % | -1.8 % | -3.5 % | -2.4 % |
| Dizzy | 8 | Male | FDA Virtual Family Project [15] | 2.7 % | 2.5 % | 1.1 % | -3.3 % |
| Billie | 11 | Female | IT'IS [16] | -2.3 % | 0.0 % | -1.0 % | -2.2 % |
| Louis | 14 | Male | FDA Virtual Family Project [15] | 0.1 % | -2.4 % | 2.1 % | -3.0 % |

**S5 Text. Headform representing a 2-year-old child**

To create a headform representing a 2-year-old child, we aimed to proportionally shrink either Roberta or Dizzy to the equivalent size of a 2-year-old. In comparing their respective average values for each facial aspect (S7 Table), we found their ratios to be remarkably similar—too close to distinguish. Given that Roberta involves licensing costs while Dizzy is freely available, we opted to shrink Dizzy. Using Dizzy as our base, we calculated the ratios of its average parameters to those of a 2-year-old (S8 Table). Subsequently, we uniformly reduced Dizzy's dimensions based on the global average of these four ratios, amounting to 113%.

**S7 Table. Ratio of facial parameters for Dizzy and Roberta in comparison to the average population.**

| Headform | Interpupillary | Bizygomatic breadth | Lower face height | Ear-Sellion depth |
| --- | --- | --- | --- | --- |
| Dizzy | 1.028 | 1.025 | 1.011 | 0.944 |
| Roberta | 0.982 | 0.965 | 0.965 | 0.976 |

**S8 Table. Ratio of facial parameters for Dizzy to the average parameters of a 2-year-old child.**

|  | Interpupillary | Bizygomatic breadth | Lower face height | Ear-Sellion depth |
| --- | --- | --- | --- | --- |
| Dizzy parameters /Average parameters for 2-year-old | 1.20 | 1.12 | 1.15 | 1.04 |

**S6 Text. Lot-to-lot Comparison of Filtration Efficiency and Pressure Drop**

Two distinct lots of two pediatric facemask brands were acquired and analyzed for both filtration efficiency and pressure drop. The results revealed no statistically significant differences between different lots, as indicated by the Student’s t-test (p > 0.05).


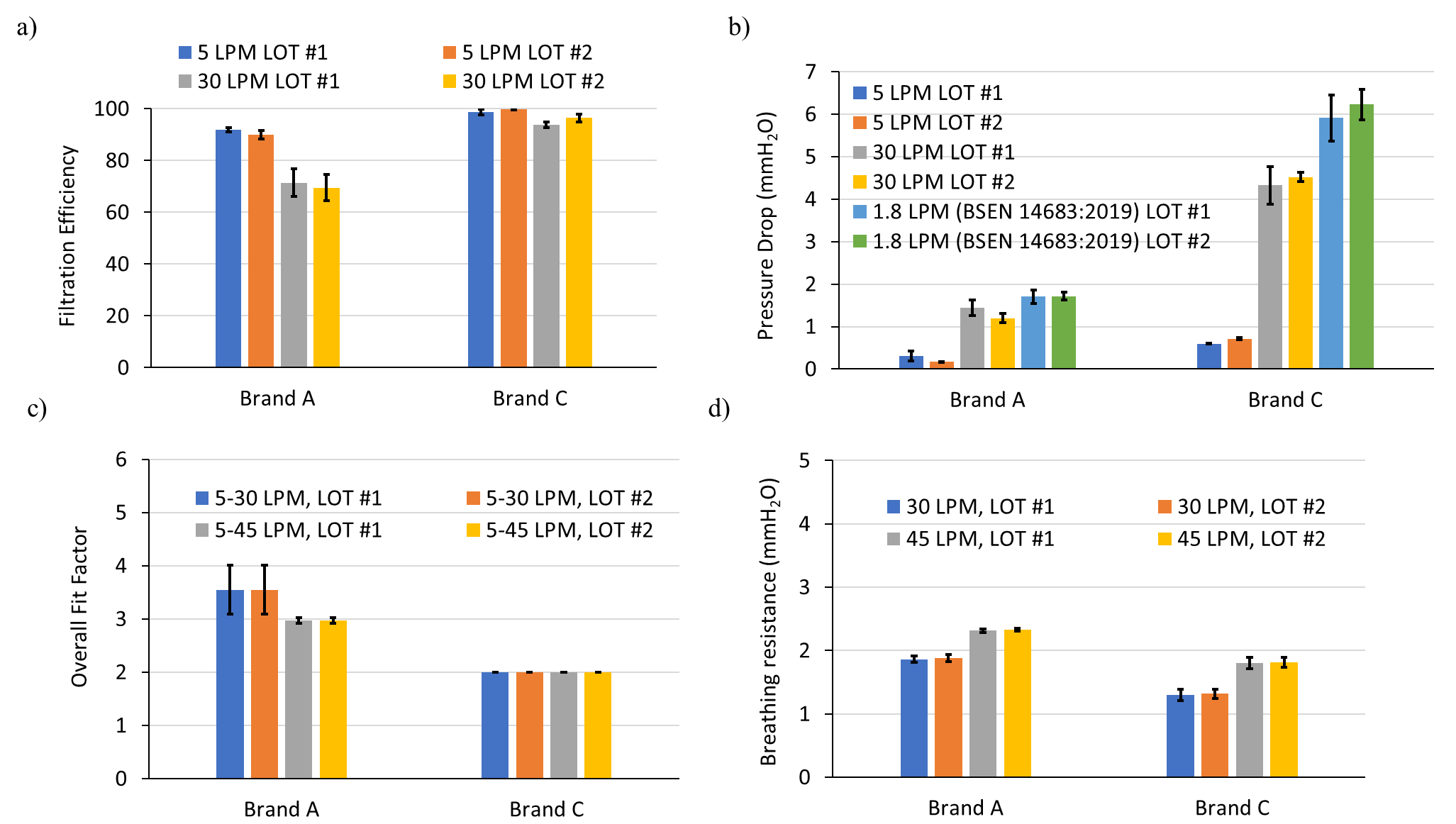


***S4 Fig.*** *Values for various lots of brands A and C; a) filtration efficiency, b) pressure drop, c) overall fit factor, and d) breathing resistance. Standard deviations shown are based on measurements made in triplicates.*

**S7 Text. Overall fit factor values for oscillatory and constant flow rates.**


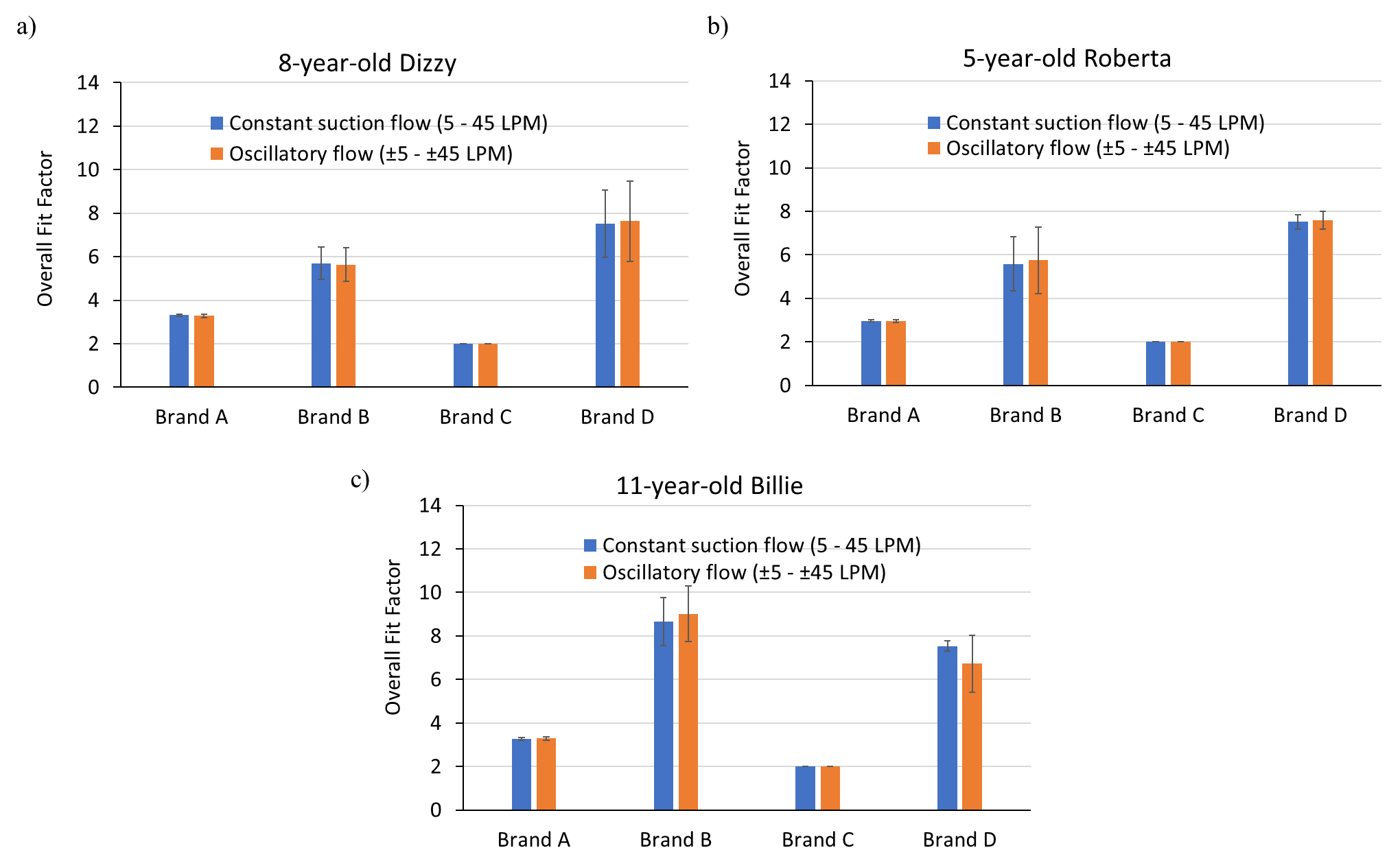


***S5 Fig.*** *Overall fit factor values for on the a) 8-year-old Dizzy, b) 5-year-old Roberta, and c) 11-year-old Billie headforms using oscillatory and constant flow rates. Standard deviations shown are based on measurements made in triplicates.*

**S8 Text. 3D printing files and Workflow for creating Pediatric Headforms**

The headforms and their related parts were printed on an EOS P 396 LPBF printer (EOS GmbH, Krailling, Germany) using polyamide 12 powder with a virgin/used mixture ratio of 50:50, slicing thickness of 0.12 mm per layer, and printing parameters recommended by EOS.

To create a pediatric headform for fit-testing and breathing resistance measurements, the following steps should be followed:

1. There are two ways to obtain the headforms file for printing: either freely from the FDA Virtual Family Project [15] and or by paying to Foundation for Research on Information Technologies in Society (IT'IS) [16]. In this study, the Dizzy (8 year old male) and Billie (11 year old female) headform is from the FDA Virtual Family Project, and hence they are being made available for use. The IT'IS headforms are licensed and, therefore, cannot be shared, and thus, are not provided.
2. The downloaded geometries were modified through several complex operations using the software Magics (Materialise). The resulting geometries are provided below (S9 Table).

**S9 Table. STL files for different parts of the 8-year-old Dizzy and 11-year-old Billie.**

| **Part** | **Purpose** | **STL file attachments** | |
| --- | --- | --- | --- |
|  |  | **Dizzy** | **Billie** |
| a) Headform with Ports on Base | Headform (S6a Fig) |  |  |
| b) Right Ear | Right ear of the headform (S6b Fig) |  |  |
| c) Left Ear | Left ear of the headform (S6c Fig) |  |  |
| d) Sample Port Adapter 0.5 in OD Acrylic Tubing | Needed for counting the inhaled aerosols (C_out_) and to create suction flow rate (S6d Fig) |  | |
| e) Metal Sample Tube Support | To enable isokinetic sampling of the inhaled aerosols (S6e Fig) |  | |
| f) Full Face 5 mm Skin Hollow with Aligner and Handle | To create the outer contour mold, face (S7a Fig) |  |  |
| g) Inner Face 5 mm Skin Solid with Aligner and Handle | To create the inner contour, face (S7b Fig) |  |  |

1. A separate geometry was created by subtracting a uniform 5 mm thickness around the face (refer to S9g Table). Since the straps of the pediatric facemasks go over the ears, not the back of the head, making skin for the back of the head was unnecessary and can be skipped to conserve material and time.
2. To create distinct components of the headform (S6 Fig), the ears were printed separately with a 1 mm subtraction (S6b and S6c Fig). A 1 mm thick layer of skin-mimicking silicone was then added around the ears [17], where the straps of the pediatric facemask loop around. Acrylic tubing (for vacuum and pressure drop measurements) and Aluminum sampling tubing with support (S6e Fig) were inserted through the holes in the mouth of the headform (The mouth sample ports should not be used outright; due to variation with 3D printing, holes in the STL files should be edited to reflect the specific clearance desired or machined after printing). Additionally, the sample port adaptor (S13d Fig) was 3D printed and affixed to the headform. Hose barb fittings were securely attached to the rear adapter using RTV silicone to facilitate the connection of vacuum and sampling hoses.
3. To fabricate all the parts listed in S9 Table, the Full Face 5 mm Skin Hollow with Aligner and Handle was printed. Then, the printed parts were coated with mold release. This printed part was then used to create a mold (S7a Fig). Making the mold requires the OUTER layer (the bigger pieces) to maintain the outer contours of the face.
4. For the mold, a box with an aligner was created, and the silicone (Mold Star 15 SLOW) was poured into the box. The OUTER sized face of the head was set into the box of silicone and cured (S7c and S7d Fig). An additional step of vacuuming could be implemented during curing to reduce bubbles within the mold.
5. Once the mold was prepared (S7e Fig), it was placed on a 5 mm smaller-sized face part of the headform.
6. Smooth-On Ecoflex 00-20 was used to create the skin layer. The Ecoflex 00-20 was poured into the mold, filling the 5 mm gap between the mold and the smaller face part (S7f Fig). Since the gap is relatively small, and the silicone can be viscous, some mold was poured into the bottom of the mold first. Then, the manikin head was placed in, and pouring would continue from the sides. Note that it is easier to trim off excess or overflow than to assess if the whole cavity is adequately filled.
7. Finally, the skin layer was carefully separated from the mold (S7g Fig), placed without being stretched, and glued onto the full 5 mm reduced headform, completing the assembly of the pediatric headform for fit-testing and breathing resistance measurements (S7h Fig).


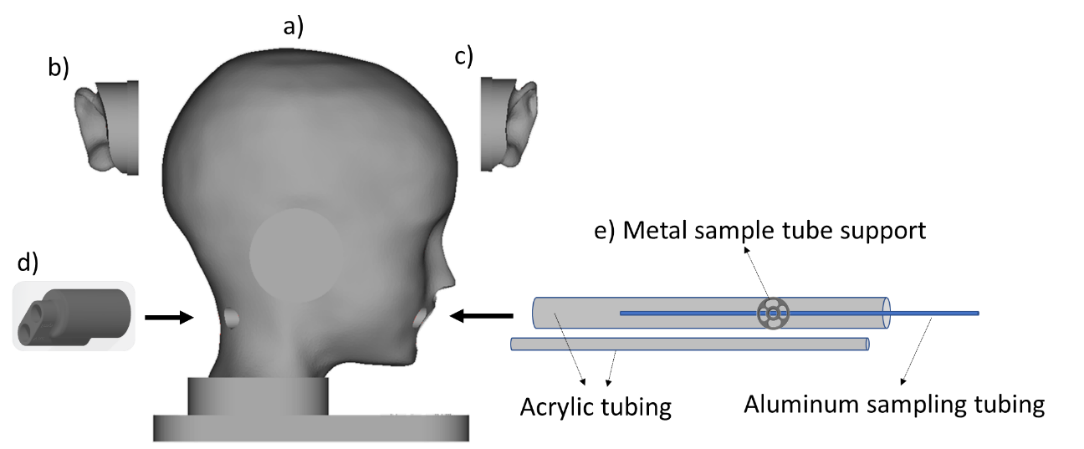


***S6 Fig.*** *Different parts of the headform with 5 mm reduced thickness. This thickness is then replenished using human skin mimicking material (S7 Fig). The acrylic tube with the smaller diameter was used as a probe for measuring breathing resistance across the facemask by connecting the tubing to a pressure gauge or transducer.*


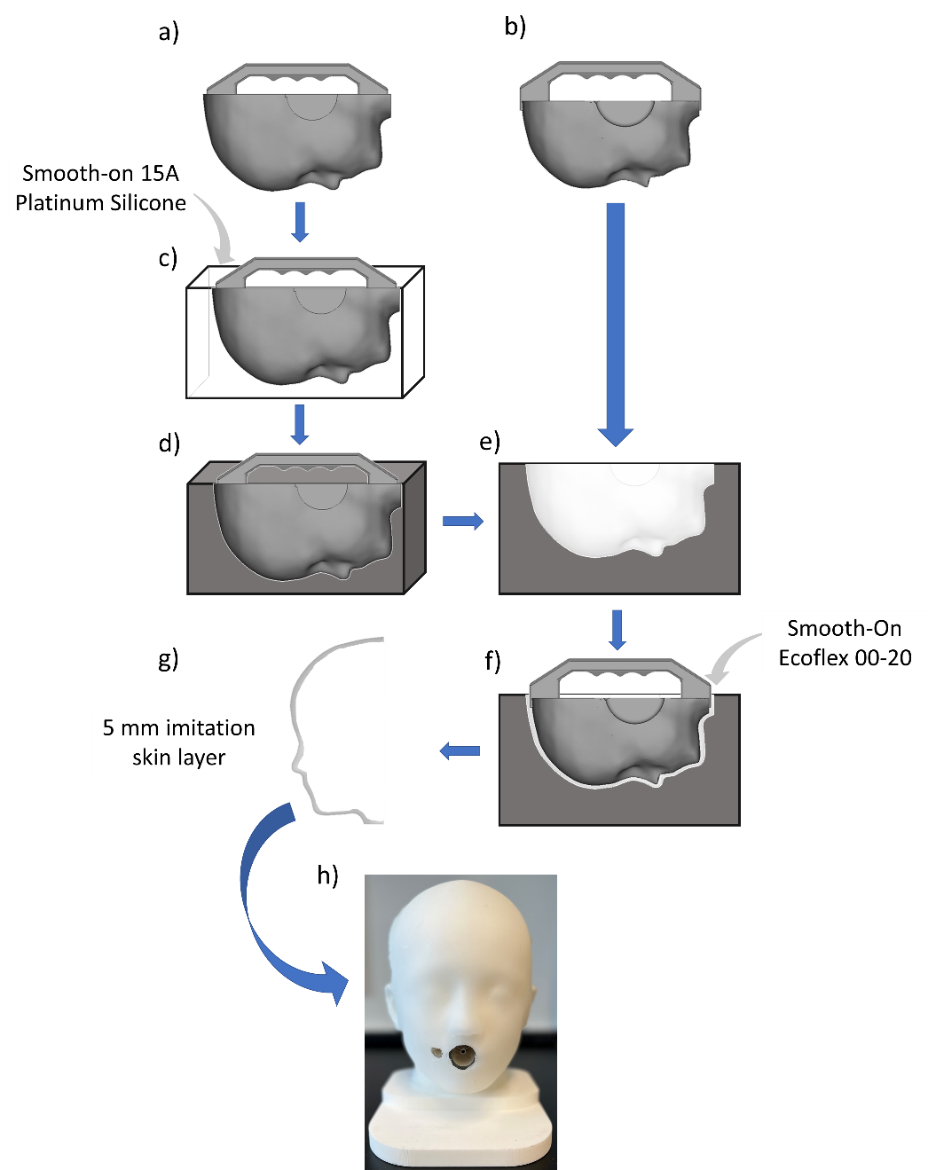


***S7 Fig.*** *Steps to create the skin layer for the pediatric manikin headform. The handles shown in figures a-b are for schematic purposes only, and although included in the STL files are not intended for printing. A user can directly purchase the handles elsewhere [18].*

**S9 Text. Operation of the PortaCount**

The PortaCount is designed for use on a person, not a headform. Consequently, many of the operations the program requests could not be performed. For our purposes, we only considered the “normal breathing” operation during the testing cycle with the manikin. The following self-check steps were executed for the PortaCount:

1. The isopropanol-soaked wick was inserted into the PortaCount.
2. Background aerosol particles were generated using the TSI 8026 particle generator with a 0.01 g/mL solution of sodium chloride.
3. The Portacount Respirator Fit Tester (TSI Inc. Model 8048) was powered up and connected to the TSI PortaCount control program (FitPro Ultra).
4. Sampling Hose Attachment during Fit Determination: Attached the sampling hose to the sampling port during the fit determination process.
5. Person, Respirator, and Protocol Selection: The process began by selecting the person, respirator, and following the outlined protocol (see S8 Fig).
6. Readiness of Fit Tester for Testing: Once the selection was made, the fit tester was prepared for testing (see S9 Fig).
7. Aerosol Concentration Measurement: The fit tester measured aerosol concentration both in the chamber and inside the facemask (refer to S10 Fig).
8. Fit Factor Determination: The fit tester determined the fit factor based on aerosol counts. Notably, for pediatric facemasks, achieving a fit factor > 100 was unattainable. Since the software reports fit factor < 100 to be a fail, consequently, after the initial exercise, PortaCount displayed "Test Complete, Failed" (see S11 Fig) and reported the corresponding fit factor. This reported value was recorded and used in calculations for measuring fit of the corresponding pediatric facemasks on the headform.
9. Restarting the Fit Test: Following a failure, the fit test could be restarted using either the same or a different flow rate.

For further details on operating the TSI Inc. 8048 model and recording data, refer to [19].


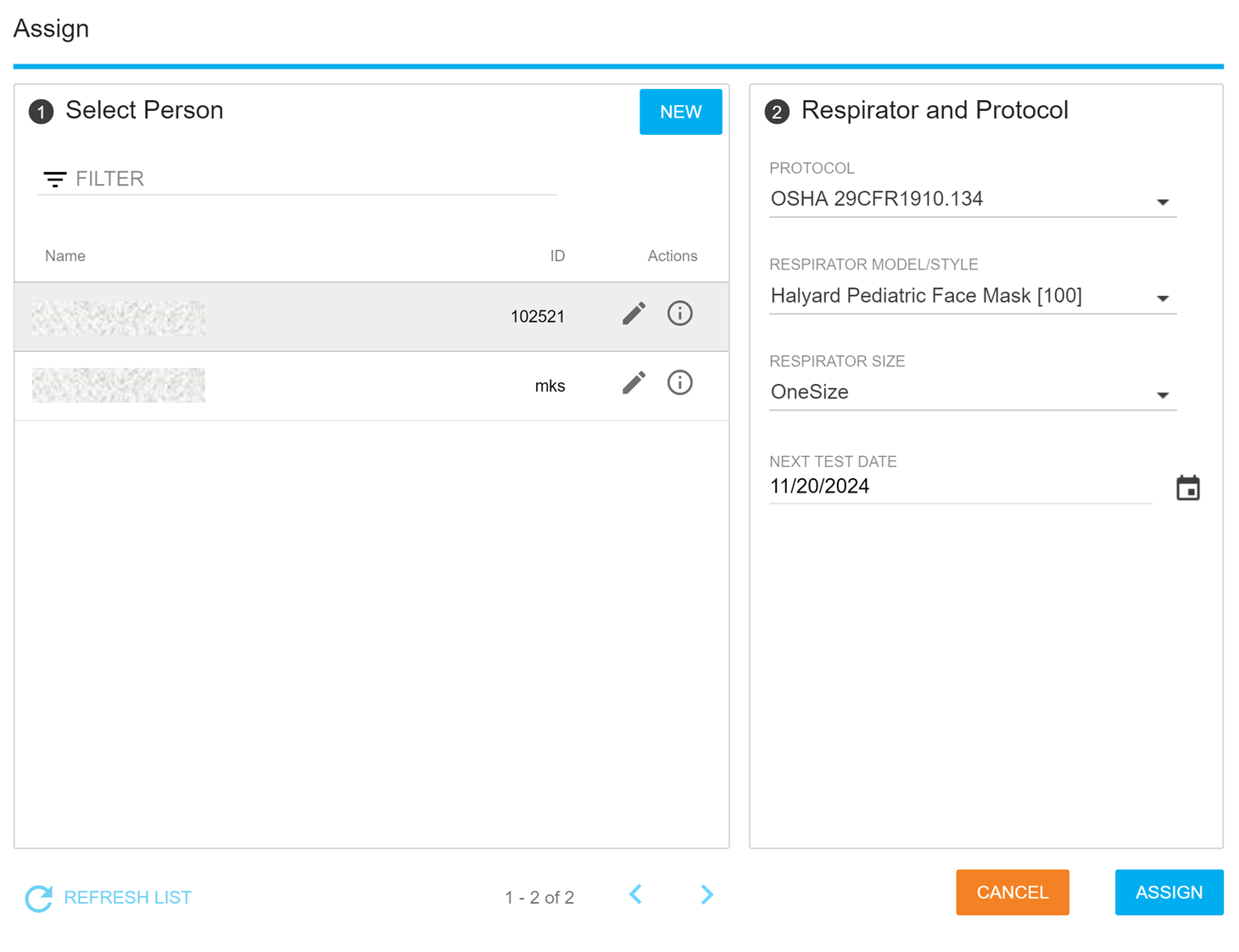


***S8 Fig.*** *Selection of Person, Respirator, and Protocol. Screenshot courtesy of TSI Inc.*

**
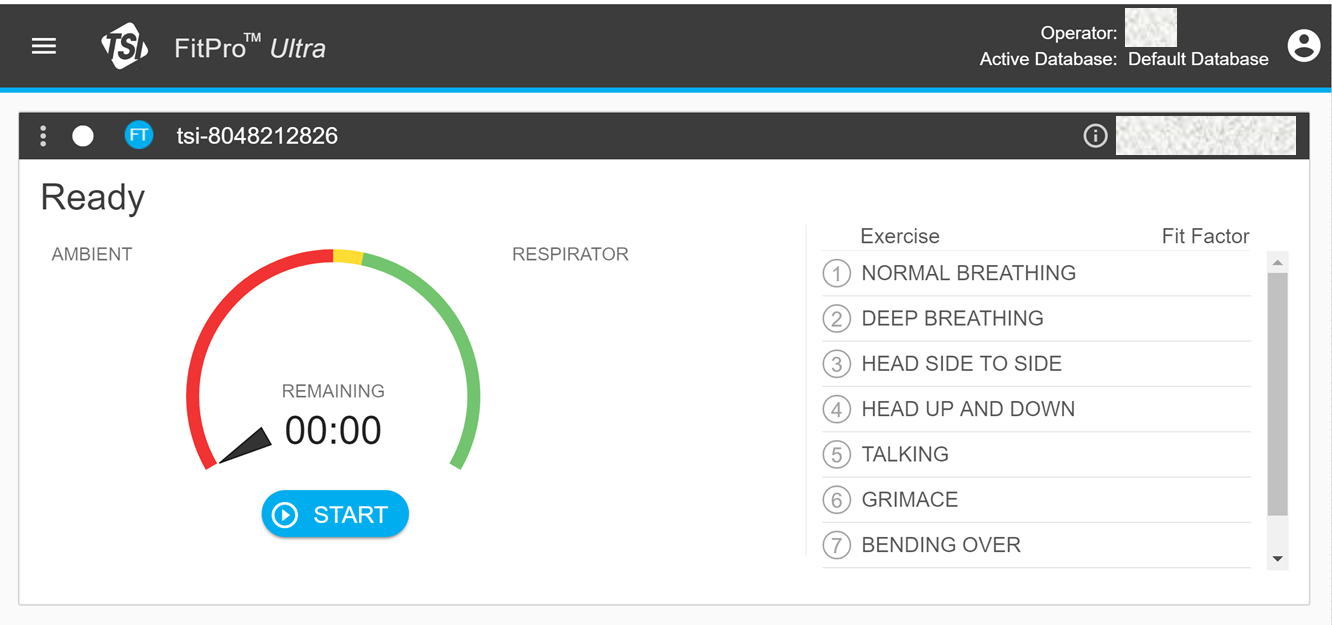
**

***S9 Fig.*** *Readiness of Fit Tester for Testing. Screenshot courtesy of TSI Inc.*


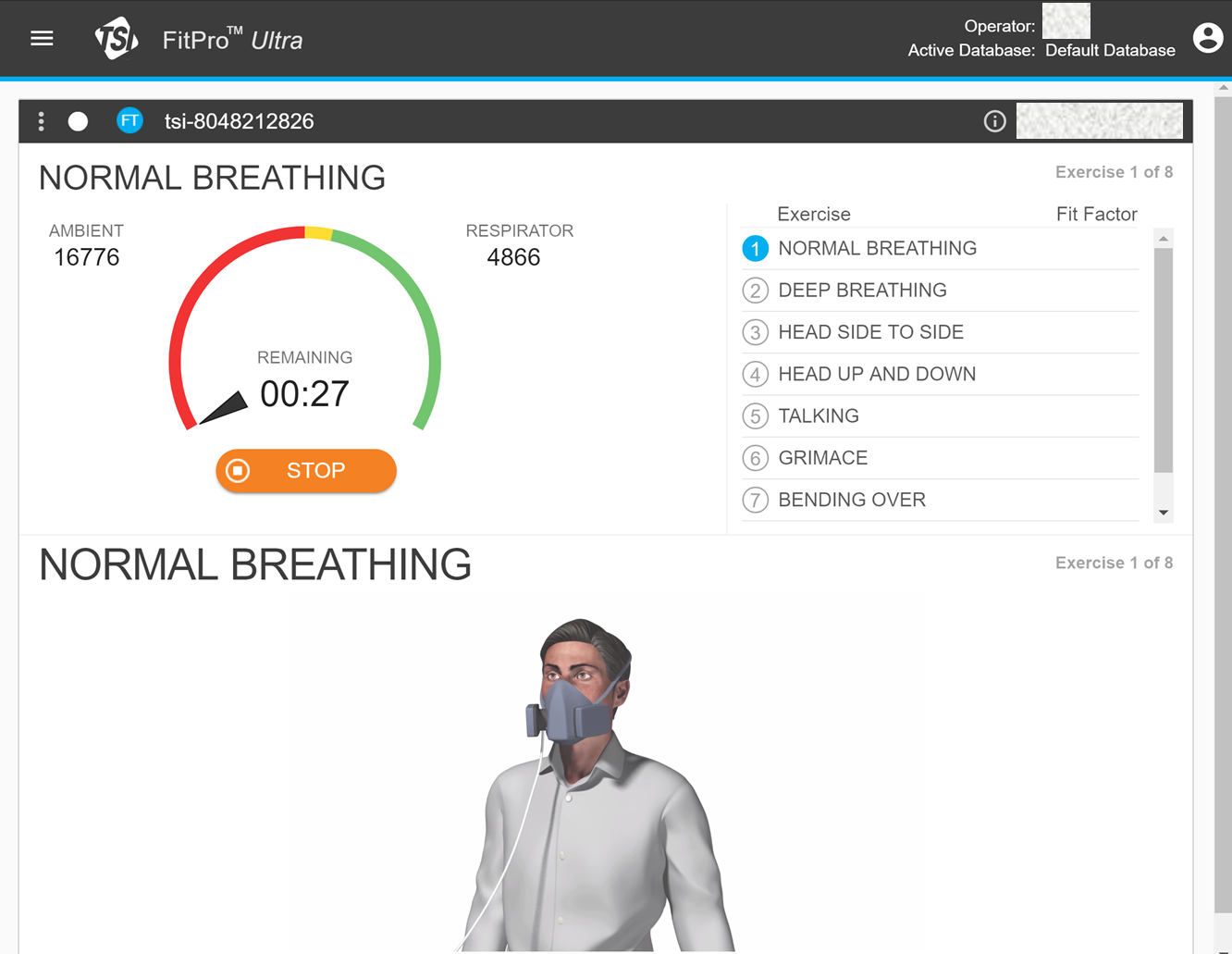


***S10 Fig.*** *The fit tester measures aerosol concentration both in the chamber and inside the facemask. Screenshot courtesy of TSI Inc.*


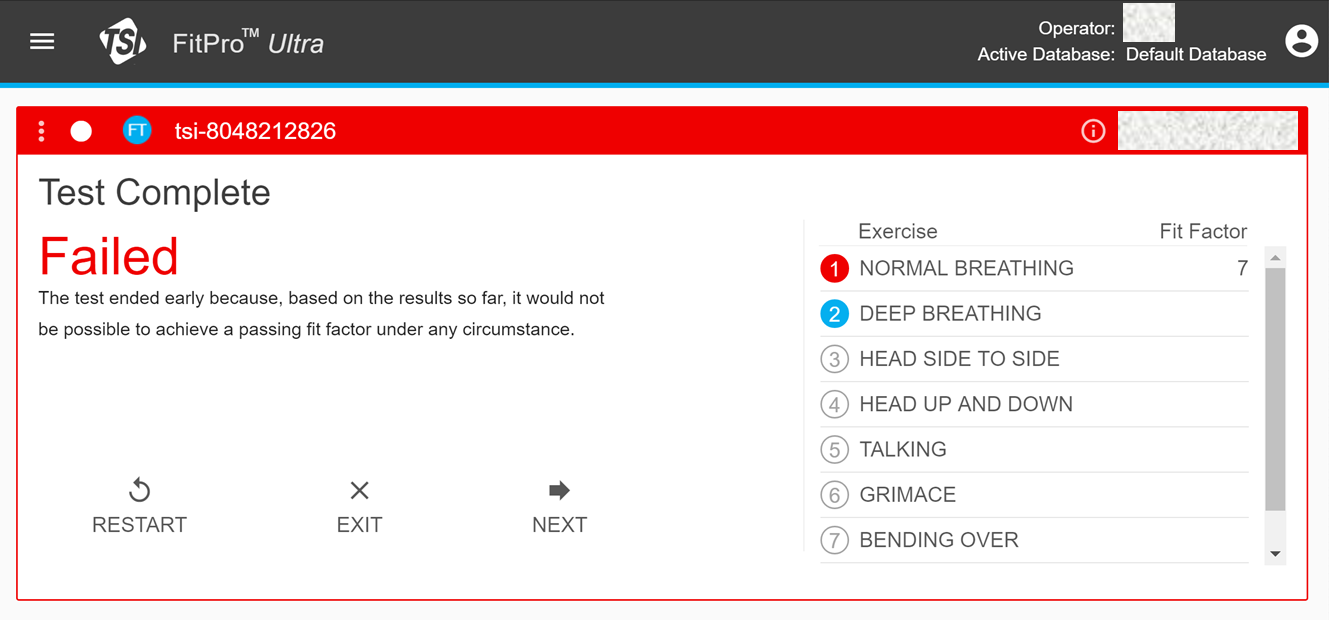


***S11 Fig.*** *PortaCount Test Complete. Screenshot courtesy of TSI Inc. The test failed as the software application is meant to assess quantitative fit testing for respirators which are meant to have Fit factor greater than 100. Note that the Fit factor shown here does not correspond to the screenshot shown in S10 Fig and is from a different experiment.*

**S10 Text. Pressure Drop Measurements**

For pressure drop measurements, we employed a method outlined in the literature [20]. We utilized highly accurate and sensitive pressure transducers that enable real-time data acquisition, subsequently averaged over time. The pressure measurement system comprised a Raspberry Pi 4 Model B (Raspberry Pi Foundation, Cambridge, UK), an ADS1115 4-channel 16-bit ADC (Adafruit, New York, NY), and two low-range differential pressure transducers with upper ranges of 2.0 inH_2_O (498 Pa) (PX165-002U, OMEGA, Norwalk, CT) and 0.25 inH_2_O (62 Pa) (PX165-0.25U, OMEGA, Norwalk, CT). For one transducer, the maximum pressure drop limit is 4 × 1.59 = 6.36 mmH_2_O, while for the transducer with the larger range, the limit is 4 × 12.7 = 50.8 mmH_2_O. If the pressure reading goes beyond the maximum limit of the first transducer, readings can be switched to the second transducer [20].

**S11 Text. Fit Testing N95 Adult Respirators on 14-Year-Old Headform**

The off-label use of pediatric facemasks for the 14-year-old headform showed a low fit of < 4 for most brands. Subsequently, fit tests were conducted using N95 adult respirators on the 14-year-old headform. Two N95 respirators from two manufacturers were chosen based on popularity, and availability. Both are certified as N95 respirators. These models had undergone FDA 510(k) clearance for marketing within the U.S. and were authorized by the FDA for decontamination purposes during the COVID-19 pandemic. S12a and S12b Fig display the overall fit factors measured at 5 and 30 LPM, and 5 and 45 LPM, along with the corresponding breathing resistance values at 5, 30, and 45 LPM.

Both brands exhibited an overall fit factor of 200, indicating a successful fit test. However, while both brands demonstrated low breathing resistance during light breathing at 5 LPM, they surpassed the threshold of 2 mmH_2_O during deep breathing at flow rates of 30 and 45 LPM, suggesting potential discomfort for the wearer. Based on these findings, while N95 adult respirators may provide protection to individuals aged 14 and above, they may not be suitable because of lack of breathability. Note that respirators may indicate a successful fit test, they are typically designed for adult use in workplaces and have not been extensively tested for widespread use in children [21] and hence their use for the pediatric population should be pursued with extreme caution and under medical and parental supervision.


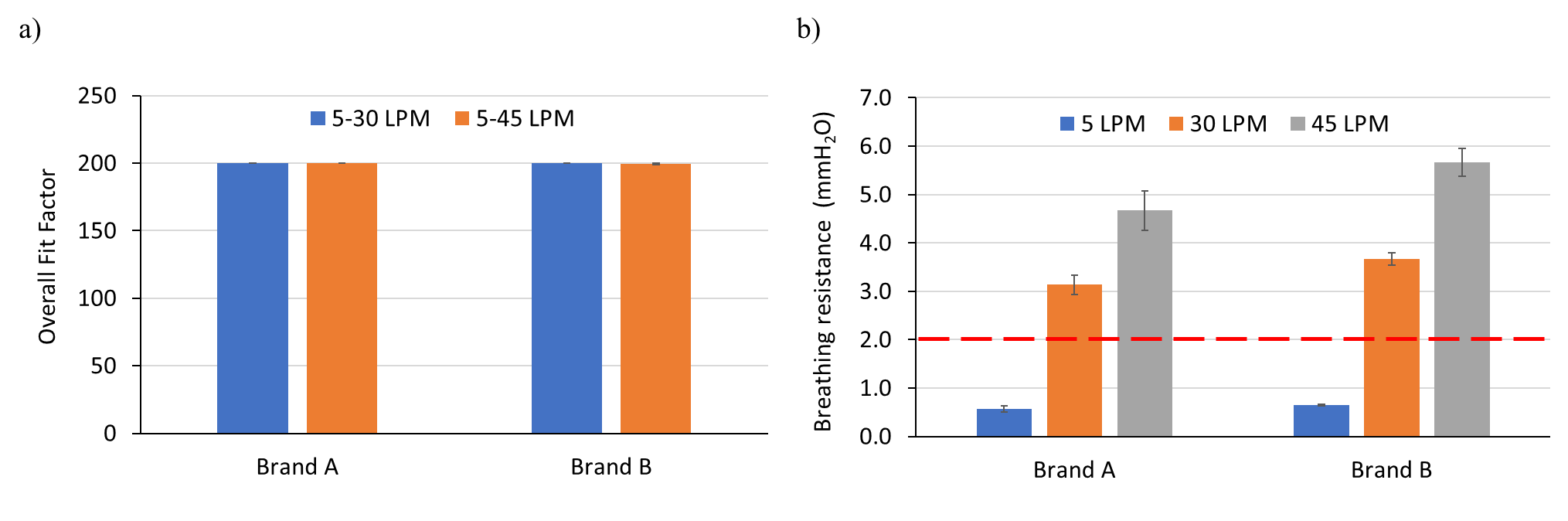


***S12 Fig.*** *Fit Testing N95 Adult Respirators on 14-Year-Old Headform; a) overall fit factors at 5-30 LPM and 5-45 LPM, b) breathing resistance values at 5, 30, and 45 LPM. Standard deviations shown are based on measurements made in triplicates.*

**References**

1. Roy, M. and C. Courtay, *Daily activities and breathing parameters for use in respiratory tract dosimetry.* Radiation Protection Dosimetry, 1991. **35**(3): p. 179-186.

2. Xi, J., et al., *Breathing resistance and ultrafine particle deposition in nasal–laryngeal airways of a newborn, an infant, a child, and an adult.* Annals of biomedical engineering, 2012. **40**: p. 2579-2595.

3. Xi, J., et al., *Simulation of airflow and aerosol deposition in the nasal cavity of a 5-year-old child.* Journal of Aerosol Science, 2011. **42**(3): p. 156-173.

4. Kesavan, J., et al., *Intranasal Deposition of Dry Particles in Anatomically Correct Physical Models of Children and Adults during Inspiratory Flow Rates Representing Sitting Awake, Light Activity, and Light and Heavy Exercise*. 2022, DEVCOM CBC-TR-1781.

5. Kwon, J.-W., *High-flow nasal cannula oxygen therapy in children: a clinical review.* Clinical and Experimental Pediatrics, 2020. **63**(1): p. 3.

6. Ejiofor, B.D., et al., *PEEP generated by high-flow nasal cannula in a pediatric model.* Respiratory care, 2019. **64**(10): p. 1240-1249.

7. Lodeserto, F.J., T.M. Lettich, and S.R. Rezaie, *High-flow nasal cannula: mechanisms of action and adult and pediatric indications.* Cureus, 2018. **10**(11).

8. Cogswell, J., *Forced oscillation technique for determination of resistance to breathing in children.* Archives of Disease in Childhood, 1973. **48**(4): p. 259-266.

9. B, S.L.A.P.-H.B., *Respiratory resistance and impedance magnitude in healthy children aged 2–18 years.* Pediatric Pulmonology, 1985. **1**(3): p. 134-140.

10. Hudgel, D.W., P. Devadatta, and H. Hamilton, *Pattern of breathing and upper airway mechanics during wakefulness and sleep in healthy elderly humans.* Journal of Applied Physiology, 1993. **74**(5): p. 2198-2204.

11. Coyne, K., et al., *Inspiratory flow rates during hard work when breathing through different respirator inhalation and exhalation resistances.* Journal of occupational and environmental hygiene, 2006. **3**(9): p. 490-500.

12. Filipović, T., *Changes in the interpupillary distance (IPD) with ages and its effect on the near convergence/distance (NC/D) ratio.* Collegium antropologicum, 2003. **27**(2): p. 723-727.

13. MacLachlan, C. and H.C. Howland, *Normal values and standard deviations for pupil diameter and interpupillary distance in subjects aged 1 month to 19 years.* Ophthalmic and Physiological Optics, 2002. **22**(3): p. 175-182.

14. Snyder, R.G., *Anthropometry of Infants, Children, and Youths to Age 18 for Product Safety Design. Final Report.* 1977.

15. Christ, A., et al., *The Virtual Family—development of surface-based anatomical models of two adults and two children for dosimetric simulations.* Physics in Medicine & Biology, 2009. **55**(2): p. N23.

16. Gosselin, M.-C., et al., *Development of a new generation of high-resolution anatomical models for medical device evaluation: the Virtual Population 3.0.* Physics in Medicine & Biology, 2014. **59**(18): p. 5287.

17. Danter, J., R. Siegert, and H. Weerda, *Ultrasound measurement of skin and cartilage thickness in healthy and reconstructed ears with a 20-MHz ultrasound device.* Laryngo-Rhino-Otologie, 1996. **75**(2): p. 91-94.

18. McMaster-Carr. Available from: https://www.mcmaster.com/products/handles/.

19. TSI. Available from: https://tsi.com/products/respirator-fit-testers/portacount%E2%84%A2-respirator-fit-tester-8048/.

20. Herman, A., et al., *A Modified Method for Measuring Pressure Drop in Non-medical Face Masks with Automated Data Acquisition and Analysis.* Journal of the International Society for Respiratory Protection, 2021. **38**(2): p. 42.

21. https://www.cdc.gov/coronavirus/2019-ncov/prevent-getting-sick/types-of-masks.html#children.
