# Supplementary material for: A retrospective characterization of pediatric facemasks marketed in the United States and implications for future designs
Source: PLoS One. 2024 Sep 19;19(9):e0307879. doi: 10.1371/journal.pone.0307879 (PMC11412539; doi:10.1371/journal.pone.0307879)
Supplement: Attachment — Submitted filename: Response to reviewers.docx [file pone.0307879.s002.docx]

**Response to Editor**

We would like to thank the editor and the reviewers for their time, detailed review and the insightful and constructive feedback. We have

- The format in which we needed to reference the supporting information figures, tables and text needed to be changed to meet journal requirements.
- We added a reference in main manuscript (reference 28) to address a comment from editor.
- We did not make any textual changes in Supporting information except for adding the description for the error bars associated with our experiments and making two figures bigger (e.g. S2 Fig) to address second reviewer’s comment on figure labeling.

Additionally, see below point by point responses to comments from the editor as well as the two reviewers below -

1. The authors do not specify the justification behind equations 1 and 2. It is unclear why the ratio of maximum permissible pressure drop to total pressure drop should be the same for adults and pediatric populations.

***Authors’ response: We would like to thank the editor for this question. Unfortunately, as we discuss in section S2 (supporting information Pages 3-4), we were not able to directly find any reference that would enable us to determine the acceptable maximum pressure drop in masks for children. However, we did find some references on N95 respirators (reference 8 in Main manuscript). Therefore, after discussion with CDC as well as our internal experts and in absence of any other appropriate metric, we ended up using equation 1, which basically enables a linear extrapolation of adult data to children. We already state this in the manuscript (page 5 of main manuscript with tracked changes) – “***we relied on extrapolating adult response for N95 respirators to come up with the maximum permissible pressure drop in pediatric facemasks.“ ***Without this starting point we would not have been able to provide any meaningful comparison or “north star” to the community on what to aim for. We also have added a Future study note (by expanding the Academia and future research section) acknowledging what type of future studies in this area are needed –***

*2. Fit-testing performed on children with various brands of facemasks for determining what nominal fit-factor may offer reasonable protection to children and to assess if the brand-to-brand differences in pressure drop across pediatric facemasks are clinically meaningful.*

***Note that equation 2 is just a reorganized form of equation 1 and is stated already in Page 5. Therefore, no additional justifications were added for equations 1 and 2.***

2. It is unclear whether differences suggested by the authors between different brands in terms of breathing resistance or filtration efficiency are practically meaningful. I would encourage authors to add a discussion on what differences in these parameters are practically relevant and will be impactful. Given that certain brand facemasks perform better than others in various experimental tests performed by the authors, can authors cite particular anecdotes suggesting the practical implications of these differences?

***Authors’ response: We thank the editor for this great question. We have added a paragraph to the discussion (in Page 14-15 of main manuscript with tracked changes) where we speculate that fit test is likely to be the most clinically meaningful. We have also added an anecdotal reference where fit and leakage was correlated with infections.***

*“While the filtration efficiency and pressure drop measurements are important for thorough characterization of a pediatric facemask, Given the lack of clinical studies conducted with pediatric facemasks, it is challenging to derive any meaningful clinical implications based on 10-20 % difference in filtration efficiency or 2-3 fold difference in pressure drop across various brands of pediatric facemasks. However, fit factor is a more meaningful metric as a fit factor of 2 versus 8 implies that a person wearing a mask with a higher fit is able to block out significantly greater number of aerosols (by a factor of 4) thus reducing risk of airborne-infection [28]. Therefore, the subsequent sections delineate the brand-to-brand performances using the metric of fit-factor.”*

**Response to Reviewer #1**

Reviewer #1: This manuscript is a good explanation of scientific research. I want to write some suggestions important for polishing the manuscript.

***Authors’ Response: We would like to thank the reviewer for their time and the valuable as well as positive feedback.***

1. The abstract needs to mention the future prospects of your studies. It would be helpful to add brief statements about practical utility, limitations, and proposed future work of this study to the abstract.

***Authors’ Response: Note that the*** [***Submission Guidelines | PLOS ONE***](https://journals.plos.org/plosone/s/submission-guidelines) ***asks that abstracts not exceed 300 words, whereas our current abstract already slightly exceeds that limit (~ 350 words). Additionally, the guideline asks that the abstract focus on the objectives, study and results and their significance and not the limitations or future studies. We are hoping that since we have a dedicated Limitations section (section # 5), that would address the reviewer’s feedback on having statements around limitations. We do sincerely value the reviewer’s proposal to add future work to the manuscript. Therefore, we have now added a new section (section 6) that discusses future studies that may be helpful to address the remaining gaps for this important topic.***

2. Figure 1 (b) contains more subdivisions. Please make sure that each subdivision (A, B, and C) is explained in the figure caption.

Authors’ Response: We apologize for the oversight. We have added the following descriptions In Figure 1 “*[A] Exploded view of the 3D printed rig for measuring pressure, [B] Computer aided design of the rig, [C] Final 3D printed rig with tubings (adapted from reference [17])”*

**Response to Reviewer #2**

Reviewer #2: In the manuscript titled, “A Retrospective Characterization of Pediatric Facemasks Marketed in the United States and Implications for Future Designs”, the authors presented three major objectives of the study which is to determine a suitable flow rate for the testing of the pediatric facemasks, define an acceptable pressure drop for the pediatric facemasks, and assess fit and breathing resistance. Even with the limitations of this study presented by the authors as well (such as the need to study pediatric population with respiratory illness and asthma, and other limitations) and some future suggestions in section 4 and 5, the study presented a good starting point to provide a workflow to evaluate the pediatric facemasks and present the need to manufacture facemasks specifically designed for various sub population that is below 4 years and above 12 years of age, which in the future might need a more detailed quantitative cutoff of the various design aspect of the pediatric facemask such as suitable flow rate, pressure drop, fit and breathing resistance.

**Authors’ Response: We truly appreciate the thoroughness of the review and the valuable constructive feedback provided.**

I have the following minor suggestions and questions.

1) In abstract, results & discussion on page 9, line 3, “retrospective assessment of four brands of legally marketed facemasks in the U.S. …” does not clarify that there are only four brands available in the U.S. for pediatric masks as mentioned later in the paper. It would be great if the authors could clarify there are only four brand available in the abstract itself. Further in the same line, “…revealed that majority of the brands have high filtration efficiency (>95%) at low flow rate 5 LPM reduces to ~ 80% at 45 LPM.” could be edited to “…5 LPM which reduces to …” with the addition of ‘which’ or a similar change.

**Authors’ Response: At the time of responding to this rebuttal, FDA has cleared more facemasks, however, we could not locate them for purchasing in US. The text in abstract and in Page 4 have been updated accordingly and also to meet the reviewer’s request. The changes to the abstract are shown below -**

A retrospective assessment of all the four brands of legally marketed facemasks in the U.S. that could be purchased, revealed that majority of the brands have high filtration efficiency (>95%) at low flow rate 5 LPM which reduces to ~ 80% at 45 LPM.

2) In the abstract, results & discussion on page 10, line 1, “Furthermore, opening the pleats of the facemasks completely results in a notable reduction in pressure drop (a 6.6-fold decrease, p=0.03).”, the authors could also add a line about the point of how the opening of pleats affected the efficiency which is later discussed in the results section.

**Authors’ Response: The studies of filtration efficiency with pleats-opened were of limited scope with us considering only two of the four brands. Besides, its implications of a < 10% drop in filtration is perhaps not as important as the significant (~ five-fold) drop in pressure. Since we had to prioritize conveying only the most important messages in the abstract, we would like to retain the liberty to not include the finding about the filtration efficiency increase with opened pleats in the abstract. We hope that the reviewer can remain open to our justification provided above.**

3) In section 2 d) Measuring Filtration Efficiency and Pressure Drop on page 13, line 3, a definition of facemask coupons would help.

**Authors’ Response: We thank the reviewer for this comment. We have now added the following sentence for added clarity –**

*These coupons were directly cut from the whole mask, with all the inner layers retained (as in a whole mask), unpleated and then recut so the cross-sectional area would be 4.9 cm^2^.*

4) In section 2 e) Method for Measuring Fit and Breathing Resistance on page 15, line 4 the authors mention the selected headforms represented pediatric individuals aged 2 to 14 years. If the age considered from 2 to 14 years is to study the off-label use as mentioned later than it would be good to clarify why the authors study 2 to 14 years here. Also, considering the use of the pediatric masks only for the FDA approved age group which is 4 to 12 years according to the authors, and considering the facial dimensions of the Roberta, Dizzy, and Billie from Figure 3, the three individuals might represent only part of the spread of general pediatric population facial features for example the Figure 3 a) suggests that Roberta and Billie are right below the average values for interpupillary distance and only Dizzy was above average, while for Figure 3 d) all three were right below average. All the three data points still fit within the deviation from the averages but for a study determining the pressure drop, and breathing resistance limits along with considering fits, a better representation of the pediatric population might be with the additional data points for the lowest possible values of facial dimensions at the lower age limit and highest possible facial dimensions at the highest age limit.

**Authors’ Response: We agree that this is a major limitation of our study and future studies can build on our current study by including additional manikin designs that capture the anthropometric features that are on the extremities of the average population. We already acknowledge some of these aspects under “Individual Variability” subsection of Limitation section (Section 5). Additionally, we have added another bullet (shown below for convenience) under *Academia and future studies* section that alludes to the need for considering other manikin designs –**

*1. Bench top studies: using 3D-printed child manikins to assess fit across a broader spectrum of diverse anthropometric features.*

5) In supplementary section figure S2 the labels are overlapping.

**Authors’ Response: We apologize for this oversight. We have updated Figure S2 with the correction as requested.**

6) In section 2 e) Method for Measuring Fit and Breathing Resistance on page 16, line 9 what are the acceptable limits of fit factor to be considered a good fit. It is later suggested on page 25 that > 10 might be a high quantitative fit but some quantitative measure while defining the fit on page 16 would be better.

**Authors’ Response: The reviewer raises an excellent point. What constitutes as a good fit is an extremely challenging question to answer because to our knowledge no studies to date have been conducted that have characterized the fit-factor of child masks before putting them to real world use and assessing the extent of protection those masks offered in curbing infections. While we can draw an analogy from respirators that have assigned protection factors of 10, and define fit factor 10 as a good fit, we recognize that accomplishing that same criterion for pediatric masks may be immensely difficult given the desirable lower pressure drop requirement for child masks. However, we do think that assessment of real-world fit, and then determining if that fit curbs infections would be a good study that can be conducted in the future. Therefore, we have now significantly expanded the Academia and Future Studies section –**

*2. Breathability: Fit-testing performed on children with various brands of facemasks for determining what nominal fit-factor may offer reasonable protection to children and to assess if the brand-to-brand differences in pressure drop across pediatric facemasks are clinically meaningful. When making such assessments it would be important to first fully characterize the mask-brand used for filtration, pressure drop, as well assess the performance of nose-bridge strips and ear loops.* Additionally, development of pediatric facemasks that can be used by those younger than 4 years, or older than 12 years old. As well as development of a stratified optimal pressure drop range for various pediatric age groups including 2-4 years, 4-12 years, as well as those above 12 years of age.

7) In section 3, page 17 line 4, “hence 45 LPM was chosen as a realistic worst-case scenario for evaluating pediatric face masks [14, 21-23].” What is the cutoff used to define ‘realistic worse-case scenario’ compared to the actual maximum flow rate. Also, a clarification as to how the realistic value for worse case scenario might be affected based on considering the limitations discussed later such as respiratory illness with current age group of 4 to 12 years, or future design for use in population with >14 years of age, would be great if added to the discussion.

**Authors’ Response: The reviewer asks a great question. Our review of existing literature found that flow rate of 45 LPM virtually covers most children up to 18 years (refer to Tables S1 and S2) thus it should cover the concern raised for children > 14 years of age. However, it does not cover the concern raised for children who may have respiratory illnesses. We have now added an additional section 6d. that covers future studies that may be helpful to determine appropriate flow rates for such cases. For this specific item the following bullet has been added -**

*c. Assessment of the difficulties around pediatric mask-donning for children who may have developmental challenges or lung diseases (e.g. asthma). These studies should also include assessment of typical flow rates for diseased lungs so the masks designed can be tested at relevant flow rates.*

8) In section 3, page 17 line 10, consider changing “~ 750 Pa or (= 76.5 mmH2O)” to “~ 750 Pa (or = 76.5 mmH2O)” with the ‘or’ within the brackets.

**Authors’ Response: Thank you for the suggestion. The suggested change has been made.**

9) In section 3, page 17 line 11, is “0.37 mmH2O/LPM × 45 LPM” considered as defined on page 13 equation 1. If yes, it would be better to clarify that.

**Authors’ Response: We do state that it came from equation (2) which is a reorganized form of equation (1). We have made it clearer as shown below –**

~~Invoking~~ Using equation (2) and assuming that the total pressure drop in adult lungs ~ 750 Pa or (= 76.5 mmH_2_O) at 105 LPM [24], and that it remains relatively unchanged at 85 LPM, the maximum permissible pressure drop in a pediatric face mask = 0.37 mmH_2_O/LPM × 45 LPM × 9.0/76.5 mmH_2_O = 2.04 mmH_2_O or ~ 2 mmH_2_O.

10) In section 4 b) 1. on page 25, the authors state, “Manufacturers may ensure that nose clips are designed for optimal fit and the clips can conform to the nose bridge adequately. This will help ensure proper fit and protection to the wearer.” It would be great to determine a quantitative measure for how ductile or malleable or a similar measure of the material of the nose clip would be required to allow a better fit as the fit/efficiency highly depended on the fit of the nose clip (specifically for brand C).

**Authors’ Response: We completely agree with the reviewer’s proposal. We have now added this as a statement in 4b (shown in red text below).**

1. Design Optimization for Fit: Manufacturers may ensure that nose clips are designed for optimal fit and the clips can conform to the nose bridge adequately. This will help ensure proper fit and protection to the wearer. *It may also be beneficial to develop a test method to assess the malleability of nose-clips and characterize the clips for better fits.*

11) In section 4 b) 3. on page 25, the authors provide quantitative measures for the manufacturers, “There is a need for developing more optimal pediatric facemasks designs with minimal breathing resistance (~ 2 mmH2O) at relatively high flow rates (45 LPM) which is lower than the breathing resistance of < 5 mmH2O described in ASTM F3502 for Barrier Face Coverings [28].” Would the 45 LPM upper limit of the flow rate to be tested change based on the age group considered as suggested in section 4 b) 2.? If yes, it would be better to clarify the age group or mention it could depend on the age group in section 4 b) 3.

**Authors’ Response: We agree with the reviewer, the flow rate would need to be a function of the flow rate. We have added a clarifying statement in section 4b)3 (page 20 of manuscript with tracked changes) and added a bullet under Academia and future research section (Page 20 of manuscript with tracked changes).**

12) Figure 4 to 8 need a statement defining what the error bars represent in the figures.

**Authors’ Response: The error bars represent experiments that have been performed at least in triplicates. The Figures 4-8 captions have now been updated reflecting that change.**
